# Glycoproteomics and Functional Characterization of Novel Variants in Siblings with ALG1-CDG

**DOI:** 10.3390/biom16091308

**Published:** 2026-09-09

**Authors:** Dongzhi Wei, Hui Wang, Senlin Peng, Yu Huang, Ganglong Yang, Weijie Dong, Xiaodong Gao, Ning Wang

**Affiliations:** 1 State Key Laboratory of Biopharmaceutical Preparation and Delivery, Institute of Process Engineering, Chinese Academy of Sciences, Beijing 100190, Chinawangh29@dmu.edu.cn (H.W.); glyang@ipe.ac.cn (G.Y.); xdgao@ipe.ac.cn (X.G.); 2 Key Laboratory of Carbohydrate Chemistry and Biotechnology, Ministry of Education, School of Biotechnology, Jiangnan University, Wuxi 214122, China; 3 College of Basic Medical Sciences, Dalian Medical University, Dalian 116044, China; wjdong@dmu.edu.cn; 4 Department of Medical Genetics, School of Basic Medical Sciences, Peking University, Beijing 100191, China; huangyu@bjmu.edu.cn

**Keywords:** congenital disorders of glycosylation, *ALG1*, splicing variant, molecular basis, glycoproteomics

## Abstract

Congenital disorders of glycosylation (CDGs) are a growing group of inborn errors caused by gene defects in glycan biosynthesis pathways. Genetic testing of two patients from the same family harboring *ALG1*-related CDG (ALG1-CDG) revealed heterozygous variations in the c.1129 A>C and c.1263+3 A>T. Although most CDGs with abnormal *N*-glycosylation can be detected by transferrin screening, Western blotting of serum transferrin from the two siblings did not reveal significant glycosylation deficiency. In this study, the biochemical phenotype and pathological molecular basis of the CDGs in the siblings were further explored. Analysis of the patients’ *ALG1* cDNA indicated that the associated heterozygous variants were c.1129 A>C (p.Met377Leu) and c.1188 T>A (p.Cys396X). Single-molecule real-time (SMRT) sequencing indicated that the variant frequencies of M377L and C396X were 50% and 36%, respectively. In addition, glycoproteomics analysis suggested that the glycoforms of transferrin were altered in the patients and that glycosylation site occupancy was decreased. It was also confirmed that *N*-glycosylation was altered in the patients’ serum transferrin. Conserved amino acid variants of yeast Alg1 were recombinantly overexpressed, purified and subjected to an in vitro quantitative activity assay. The results showed that these variants caused a decrease in enzymatic activity, suggesting pathogenicity.

## 1. Introduction

**Figure 1:**
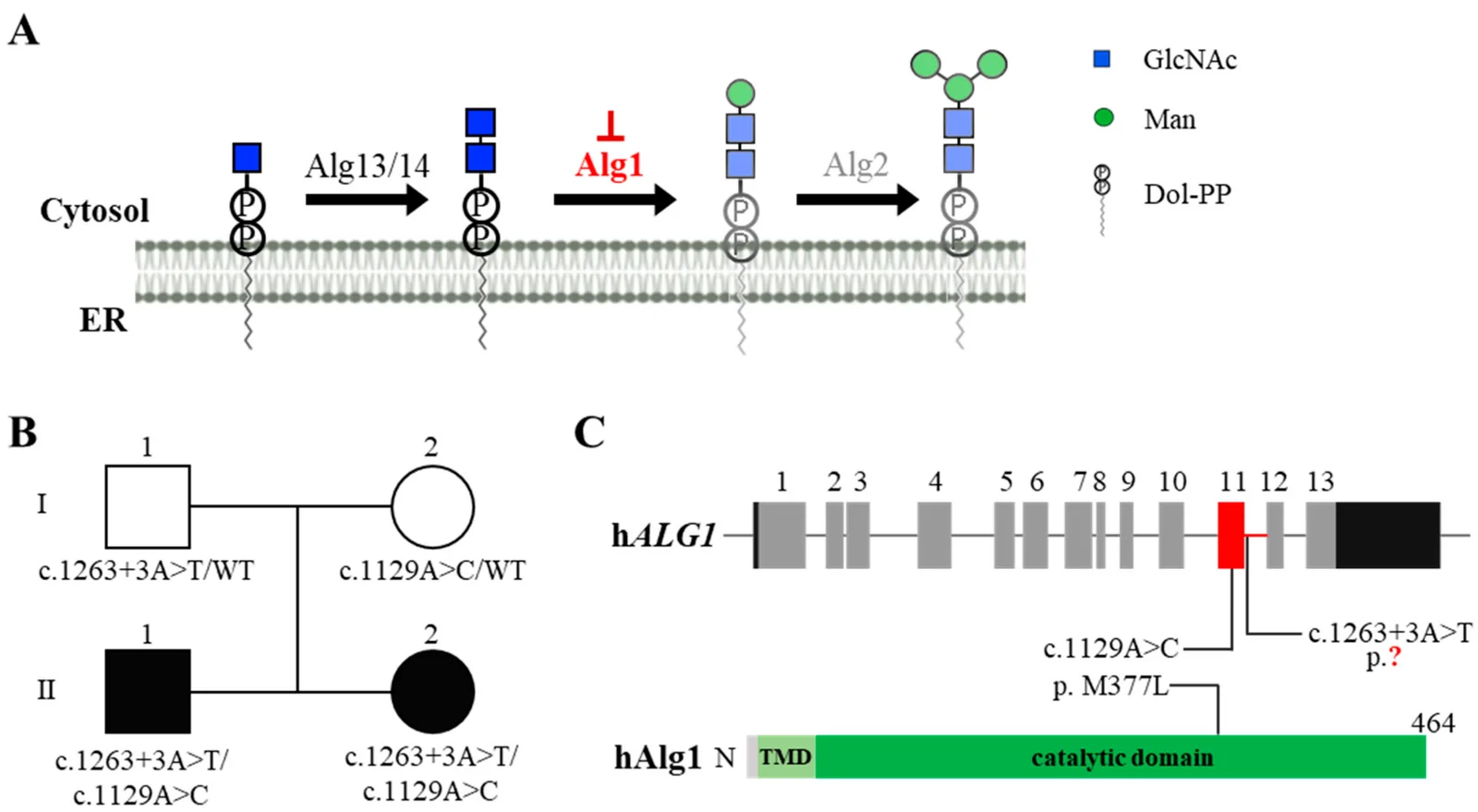
Schematic representation of the pathway associated with the Alg1 enzymatic step and the *ALG1* variants identified in the pedigree. (**A**) Involvement of Alg1 in the LLO biosynthesis pathway. Blue square: *N*-acetylglucosamine (GlcNAc), green circles: mannose (Man), and wavy lines: dolichol (Dol). The *ALG1*-dependent step is highlighted in red with a “┴” indicating the deficiency. (**B**) Pedigree of the family with ALG1-CDG. Individuals II-1 and II-2 are the ALG1-CDG patients studied in this work. ALG1-CDG patients are represented by filled black symbols. (**C**) Locations of the *ALG1* variants identified in this study, which were c.1129A>C and c.1263+3A>T.

Congenital disorders of glycosylation (CDGs) are a growing group of genetic diseases caused by defects in genes that encode related enzymes in glycan biosynthesis pathways [1,2]. Since the first description in the 1980s, over 100 different types of CDGs have been reported, and most reports describe *N*-linked glycosylation-related CDGs [3,4,5]. *ALG1* encodes an essential β-1,4-mannosyltransferase (Alg1, EC:2.4.1.142), which localizes to the cytoplasmic side of the endoplasmic reticulum and catalyzes the transfer of the first mannose (Man) to dolichyl-pyrophosphate-GlcNAc2 (GlcNAc2-PP-Dol) to produce the lipid-linked oligosaccharide (LLO) precursor Man1GlcNAc2-PP-Dol in the *N*-glycan biosynthesis pathway (Figure 1A) [6,7,8]. In humans, *ALG1* has 13 exons and codes for a 464 amino acid protein, whose variants cause abnormal assembly of *N*-glycans on glycoproteins, leading to ALG1-CDG [4,9,10,11] which was first reported in 2004 as CDG-Ik [9,12,13].

The number of patients suffering from ALG1-CDG, the third most common type of CDG, is rapidly increasing worldwide, with approximately 100 cases and more than 50 variants reported [14,15,16,17]. Patients with ALG1-CDG usually present with neurological abnormalities, such as epilepsy, psychomotor retardation, microcephaly, and other multisystem diseases, which poses challenges for clinical diagnosis [10,18,19,20,21,22]. In 2021, 2 siblings with *ALG1* deficiency were reported in China as the first cases of ALG1-CDG with infantile spasms, both of whom were from the same family (II-1 and II-2, Figure 1B) [23]. Whole-exome sequencing indicated that the patients harbored compound heterozygous variations, c.1129 A>C and c.1263+3 A>T, in the *ALG1* gene, which were inherited from their mother and father, respectively (Figure 1C). In this work, we report the genetic characteristics and molecular mechanism underlying the symptoms of these two siblings, confirming that the novel variant caused *ALG1* deficiency. Although the patients did not exhibit significant glycosylation deficiency, their glycoforms and site occupancy were altered. Furthermore, the *ALG1* mutants exhibited significantly reduced activity in vitro. These observations confirm that pathogenic variations, including a novel splicing variant, lead to *ALG1* deficiency, an inherited disorder associated with *N*-glycosylation.

## 2. Materials and Methods

### 2.1. Patients

Gene sequencing of the two siblings indicated they had the variant c.1129 A>C (p.Met377Leu) and the splicing variant c.1263+3 A>T in the *ALG1* gene, which were inherited from the parents. Plasma samples were available for two siblings and their father. Plasma samples from a known ALG1-CDG patient and a healthy individual from our own plasma databank were used as controls.

### 2.2. Genetic Analysis

Total RNA was extracted from control and patient blood samples using RNAiso Plus reagent (Takara Biotechnology (Dalian) Co., Ltd., Dalian, China; Cat. No. 9108) following the manufacturer’s instructions. The RNA was placed on ice and immediately reverse-transcribed by reverse transcriptase (Takara Bio, China). Reverse transcription polymerase chain reaction (RT-PCR) was performed at 37 °C for 20 min. The synthesized cDNA was used as a template, and the coding sequence of human *ALG1* was amplified by PrimeSTAR^®^ GXL DNA Polymerase (Takara Bio, China). PCR was performed following the manufacturer’s protocol, with primers P1F and P1R (Supplementary Table S1) at 0.5 µM, an annealing temperature of 58 °C for 15 s, and an extension temperature of 68 °C for 110 s for 32 cycles.

Amplified cDNA was cloned into plasmid pMD-18T by TA cloning. Seven single colonies were selected from each plate for colony PCR, and the PCR products were verified by agarose gel electrophoresis. Clones were sequenced using standard primers M13F and M13R (Supplementary Table S1), following standard procedures, via an automated DNA sequencer (GENEWIZ, Suzhou, China). Amino acid sequence alignments were performed using the Sequence Manipulation Suite (http://www.bioinformatics.org/sms2/color_align_cons.html).

The variant frequency was determined via single-molecule real-time (SMRT) sequencing. Briefly, PCR products were purified using a SanPrep Column PCR Product Purification Kit (Sangon Biotech, Shanghai, China; Cat. No. B518141) and subjected to quality control. The qualified PCR products were submitted to GENEWIZ (China) for SMRT sequencing. The sequencing library was constructed by ligating the purified DNA fragments with adapters according to the standard SMRT sequencing workflow [24]. After sequencing and primary data processing, circular consensus sequences (CCSs) were generated and aligned to the reference template. The variant frequency was calculated based on the proportion of reads containing the corresponding variant among the total aligned CCS reads.

### 2.3. Western Blot Analysis of Serum Transferrin

Five serum samples (10 µL each) from the two siblings (D and S), the father (I-1, F), a known ALG1-CDG patient (P), and a healthy individual (male, in his twenties, C) were analyzed. The ALG1-CDG patient sample (P) was used as a positive control for hypoglycosylation, whereas the healthy individual sample (C) served as a negative control representing the normal TRF glycosylation pattern.

For sample pretreatment, all five serum aliquots were initially treated with Albumin Erasin Reagent (Sangon Biotech, China) to remove albumin (ALB), after which the Spin Albumin and IgG Erasin Kit (Sangon Biotech, China) was used to further remove IgG. ALB- and IgG-depleted samples were separated by 8% sodium dodecyl sulfate polyacrylamide gel electrophoresis (SDS‒PAGE) gel and transferred onto polyvinylidenedifluoride membranes, after which proteins were detected by enhanced chemiluminescence (ECL). Immunoblotting was performed using an anti-transferrin antibody (rabbit monoclonal, 1:3000, Sigma-Aldrich, St. Louis, MO, USA)) as the primary antibody and goat anti-rabbit IgG-HRP (1:5000, TransGen Biotech, Beijing, China) as the secondary antibody.

### 2.4. Isoelectric Focusing (IEF) Analysis of Serum Transferrin (TRF)

The human TRF standard (STD) and serum from CDG patients were analyzed by two-dimensional electrophoresis (2-DE). The first-dimensional separation was performed by isoelectric focusing analysis using an immobilized pH gradient (IPG) strip (7cm, pH 4–7, Bio-Rad, Hercules, CA, USA). The patient’s serum sample was pretreated using the Spin Albumin and IgG Erasin Kit (Sangon Biotech, China) to reduce the impact on low-abundance proteins. The IPG strip was placed in the strip holder, and hydration buffer containing 8 M urea, 4% 3-[(3-Cholamidopropyl) dimethylammonio]-1-propanesulfonate (CHAPS, Sangon Biotech, China), 65 mM dithiothreitol (DTT, Sigma-Aldrich, USA), 0.2% (*w*/*v*) Bio-Lyte (Bio-Rad, USA), 0.01% bromophenol blue (BPB) and serum sample was added to allow the IPG strip to rehydrate overnight; the sample entered the gel via absorption. The Ettan IPGphor III IEF system (Cytiva, Marlborough, MA, USA) was used for desalting, voltage ramping, and focusing using the specified voltage and time parameters. Then, the IPG strip was equilibrated twice using equilibration buffer consisting of 6 M urea, 75 mM Tris-HCl (pH 8.8), 29.3% (*v*/*v*) glycerol, 2% SDS (*w*/*v*), and 0.01% BPB. DTT was added for the first equilibration, and iodoacetamide (IAM, Sigma-Aldrich, USA) was added for the second. The second-dimensional separation by SDS-PAGE was performed on an 8% polyacrylamide gel, followed by Coomassie brilliant blue staining.

### 2.5. Glycoproteomic Analysis of Transferrin (TRF) by LC‒MS/MS

ALB- and IgG-depleted samples from the healthy control (C), father (F), and two siblings (D and S) were analyzed by LC-MS/MS, where the healthy control sample served as the reference for comparison of TRF glycosylation profiles. Meanwhile, these ALB/IgG-depleted samples (C, F, D and S) were separated by 8% SDS-PAGE and stained with Coomassie brilliant blue. The TRF band on the gel was excised into small pieces and washed 5 times with ddH_2_O. The precipitate was destained with 100 mM NH_4_HCO_3_: acetonitrile (ACN, Sigma-Aldrich, USA) (1:1, *v*/*v*), dehydrated with ACN, and incubated with 100 mM NH_4_HCO_3_ and 10 mM dithiothreitol (DTT, Sigma-Aldrich, USA) at 56 °C for 45 min. The gel pieces was then alkylated with 10 mM iodoacetamide (IAM, Sigma-Aldrich, USA) in the dark for 50 min at room temperature. The proteins were digested in-gel with sequencing grade trypsin (1:40, *w*/*w*, Beijing Shengxia, Beijing, China) over night at 37 °C, and the resulting glycopeptides were dissolved in 5% formic acid (FA, Sigma-Aldrich, USA) in ACN and lyophilized. The glycopeptides were resuspended in 1% FA and loaded onto a C18-tip column prepared from yellow pipette tips packed with 3 layers of C18 membrane (SPE Disks, CDS Analytical LLC, Oxford, PA, USA). After washing 4 times with 1% FA (100 µL each), the glycopeptides were eluted with 0.1% FA in 50% ACN (200 µL). The glycopeptide concentration was measured using a NanoDrop 2000 (Thermo Scientific, Waltham, MA, USA).

For each injection, 2 µg of lyophilized glycopeptide was resuspended in 0.1% FA in 2% ACN and subjected to MS analysis using a high-resolution Orbitrap Fusion Lumos mass spectrometer (Thermo Scientific, USA). The samples were separated on an EASY-nLC 1200 system (Thermo Scientific, USA) equipped with an internal RSLC C18 column (75 µm × 25 cm) using the following conditions: eluent A, 0.1% FA; eluent B, 90% ACN/0.1% FA. Spectra were collected from *m*/*z* 350 to 2000 at a resolution of 60,000, followed by data-dependent HCD MS/MS; the HCD collision energy was 35% and the stepped collision energy was 5% [25,26]. The acquired MS/MS data were subsequently analyzed using pGlyco3 (pFind Lab, Institute of Computing Technology, Chinese Academy of Sciences, China) and analyzed by Fragpipe (version 18.0) powered by MSFragger (version 3.5, Nesvizhskii Lab, Department of Pathology, University of Michigan Medical School, USA), Philosopher (version 4.4.0, https://philosopher.nesvilab.org/) and IonQuant (version 1.8.0) [27,28]. The TRF FASTA file (P02787.FASTA) was used as the protein database (pGlyco and MSFragger), and pGlyco-N-Human.gdb, which contains 2922 glycoforms (last updated: 15 June 2021), was used as the searching database in pGlyco [28]. The search parameters were a precursor mass tolerance of 10 ppm and a fragment tolerance of 20 ppm. The false discovery rate (FDR) for glycopeptides was 0.01.

### 2.6. Quantitative Activity Assay of Recombinant Alg1

The expression plasmid pET28-His6-Alg1ΔTM, encoding His6-tagged *S. cerevisiae* Alg1 lacking the 34-amino-acid transmembrane domain (*Sc*Alg1ΔTM), was constructed by PCR amplification of a 1245 bp *ALG1* fragment which was inserted into the *Nhe*I and *Xho*I sites of pET28 (Merck, Burlington, MA, USA) in frame with an *N*-terminal His6-tag. The Met366Leu (M366L) mutant was introduced by overlap extension PCR using mutagenic primers SMF and SMR (Supplementary Table S1), and verified by DNA sequencing (GENEWIZ, China). For protein expression, plasmids were transformed into *E. coli* Rosetta (DE3) cells (Merck, USA), and transformants were selected on LB agar plates containing kanamycin (50 µg/mL, Sangon Biotech, China). Single colonies were cultured in Terrific-Broth (TB) at 37 °C to an OD_600_ of 0.8–1.0. The culture was cooled to 16 °C and induced with 0.1 mM IPTG (Sangon Biotech, China), followed by shaking overnight at 16 °C. Recombinant proteins were extracted and purified with a HisTrap HP affinity column (Cytiva, USA) as described previously [7]. The expression level and purity of the proteins were confirmed by Western blotting and SDS-PAGE, respectively. Immunoblotting was performed using an anti-His mouse antibody (TransGen Biotech, China) as the primary antibody and anti-mouse IgG (H+L) HRP Conjugate (TransGen Biotech, China) as the secondary antibody. Protein samples were boiled for 5 min at 100 °C before loading. Protein concentrations were measured using a bicinchoninic acid assay kit (Sangon Biotech, China).

The lipid-linked acceptor GlcNAc2-pyrophosphate-phytanyl (GlcNAc2-PP-Phy) was chemically synthesized as previously reported [7]. Unless otherwise specified, all reagents were purchased from Sangon Biotech. The standard reaction mixture (final volume, 0.05 mL) contained the following components: 14 mM MES/NaOH (pH 6.0), 4 mM potassium citrate, 10 mM MgCl_2_, 0.05% NP-40, 1 mM GDP-Man, 50 µM GlcNAc2-PP-Phy and 1.6 µg/mL recombinant *Sc*Alg1. The mixture was incubated at 30 °C for 30 min and then heated at 100 °C for 2 min. Glycans were released by adding 40 mM HCl and heating at 100 °C for 1 h. The water-soluble fraction was desalted by solid-phase extraction using 1 mL of Supelclean ENVI-Carb slurry (Sigma-Aldrich, USA) equilibrated with 2% ACN. Oligosaccharide-containing fractions were eluted with 25% ACN and lyophilized. Desalted oligosaccharides were analyzed on a Dionex Ultimate 3000 UPLC (Thermo Scientific, USA) equipped with a Waters Acquity UPLC BEH Amide column (1.7 µm, 2.1 × 100 mm). The elution condition was as follows: eluent A, ACN; eluent B, H_2_O; gradient: 0–2 min, 20% B; 2–15 min, 20–50% B; 15–18 min, 50% B; flow rate, 0.2 mL/min. ESI-MS data of the eluate were measured on a TSQ Quantum Ultra (Thermo Scientific, USA) in the mass range of 400–1600 (*m*/*z*, positive mode). The oligosaccharide transfer rate was quantified by calculating the peak intensity by LC-MS using Xcalibur software (version 2.0, Thermo Scientific, USA).

## 3. Results

### 3.1. Identification of Alternative ALG1 Variants

**Figure 2:**
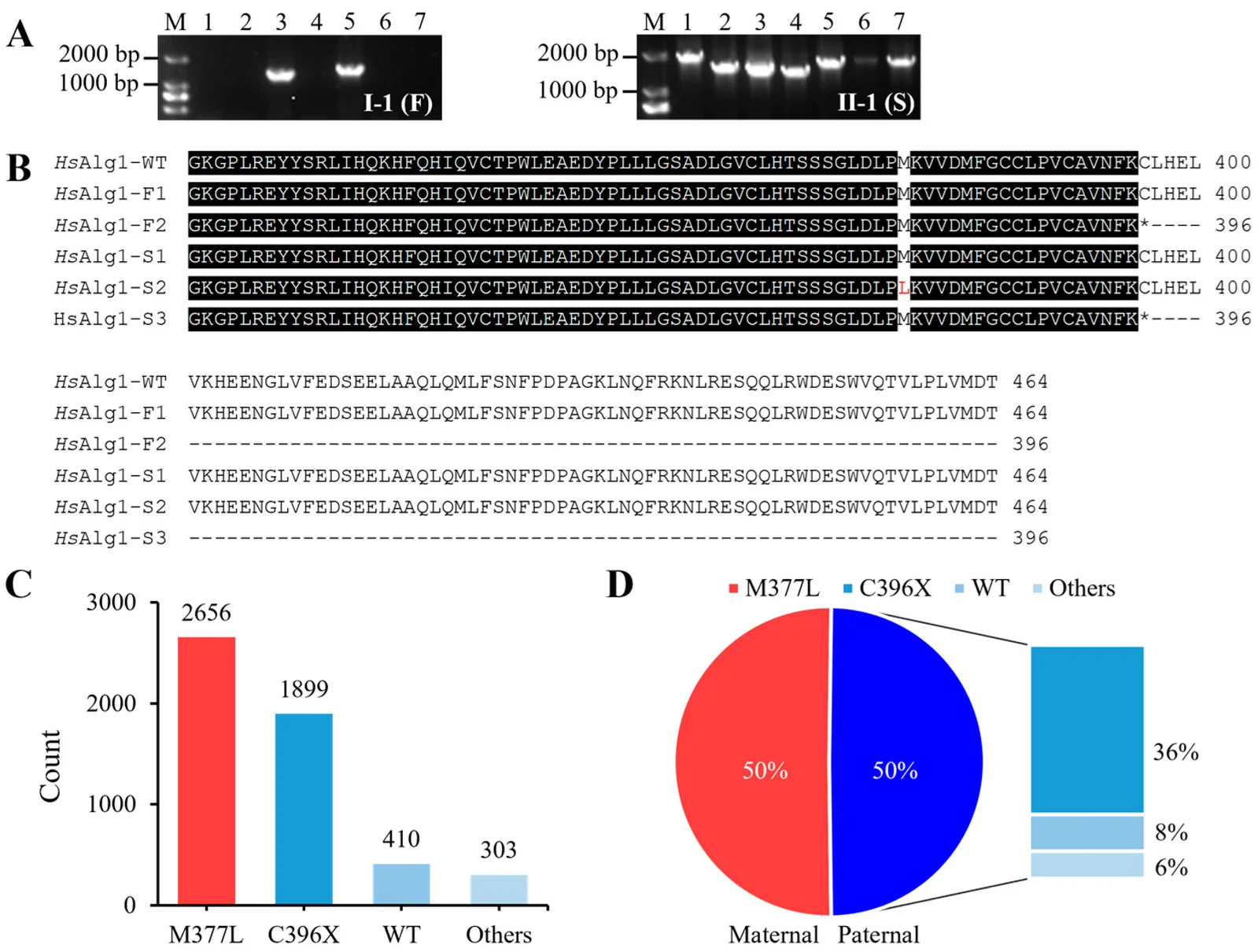
Genetic analyses of variants identified in patients. (**A**) Nucleic acid gel of PCR samples. “F” indicates the patients’ father (I-1); “S” indicates the brother in the siblings (II-1); “M” indicates DNA Marker; numbers “1–7” indicate colony PCR products. (**B**) Alignment of amino acid sequences of *Hs*Alg1, father’s Alg1 (*Hs*Alg1-F1 and *Hs*Alg1-F2) and son’s Alg1 (*Hs*Alg1-S1, *Hs*Alg1-S2 and *Hs*Alg1-S3). Conserved amino acids are shaded. Asterisk (*) indicates the stop amino-acid position. (**C**) Counts of patient *ALG1* cDNA variants. There were 2656 M377L sequences, 1899 C396X sequences, 410 WT sequences and 303 other sequences. “Other” indicates the error sequences caused by amplification or other reasons. (**D**) Distribution of mutant sequences. The M377L variant inherited from the maternal allele accounted for 50.42% of the total sequences, and the C396X variant inherited from the paternal allele accounted for 36.05% of the total sequences.

RNA extracted from blood was reverse-transcribed to synthesize cDNA, which was cloned into plasmid pMD-18T. The PCR products were analyzed by agarose gel electrophoresis (Figure 2A), and the target bands were subjected to sequencing. As shown in Figure 2B, cDNA from the patient’s father (F) had one variant; namely, c.1188 T>A (p.Cys396X, C396X; X indicates the stop codon). cDNA from the son (S) contained heterozygous variants c.1129 A>C (p.M377L) and c.1188 T>A (p.C396X), which were inherited from the mother and father, respectively. Because cDNA is transcribed from mRNA, which contains only exons, the C396X variant was likely generated by the c.1188T>A variant at the splice sites.

### 3.2. Quantification of Transcript-Specific ALG1 Expression in Patient Blood Samples

Patient cDNA was analyzed by SMRT sequencing to determine the variant frequencies. The amplified cDNA reached a concentration of 35 ng/µL and a total amount of 3360 ng, meeting the requirements for library construction. The library was subjected to amplicon sequencing, and the CCS data were aligned to the reference template. Among the 5268 amplicons, approximately half of the *ALG1* sequences (2656 counts) were M377L inherited from the mother (Figure 2C,D). The other half of *ALG1* (2612 counts) was affected by the intronic variant from the father, which produced the C396X variant (1899 counts) and the wild-type sequence (410 counts). Because the c.1188 T>A variant is located at a splicing site next to exon 11, it also introduced other aberrant sequences (303 counts). It was calculated that 36.05% of the patients’ *ALG1* contained the C396X mutant (Figure 2D), whereas 7.78% were wild-type (WT).

### 3.3. Plasma TRF Analysis Reveals the Nearly Normal N-Glycan Pattern in Two Siblings

**Figure 3:**
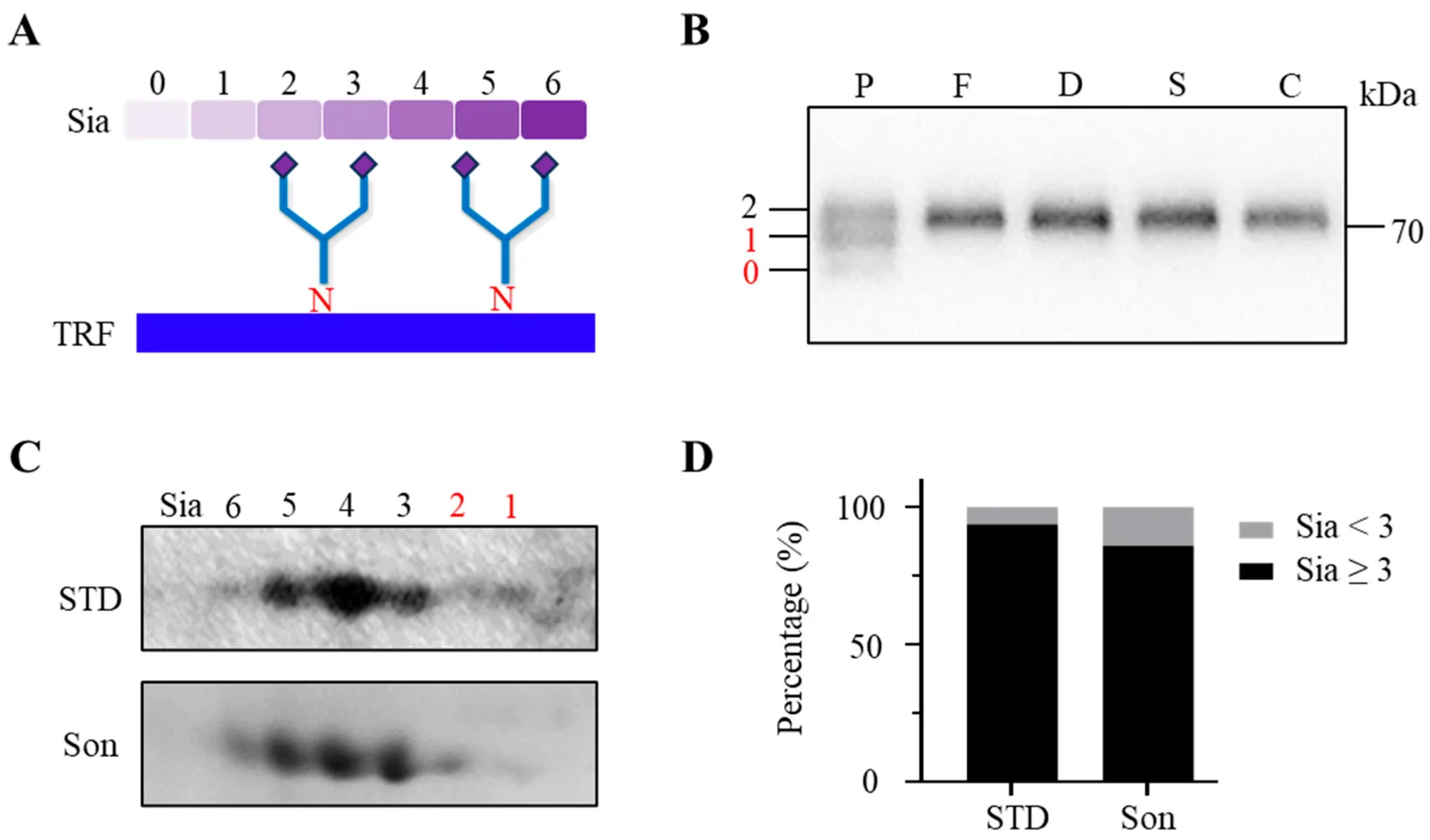
Glycoform analysis of the TRF. (**A**) Human TRF has two *N*-glycosylation sites, each bearing a complex-type glycan with up to three terminal sialic acid residues, resulting in a total of 0–6 sialic acids per molecule. (**B**) Western blot of serum TRF from a known ALG1-CDG patient (P), the father (I-1, F), the daughter (II-2, D), the son (II-1, S) and a healthy control (C). Numbers (2, 1 and 0) indicate the quantity of complete *N*-glycans on serum transferrin. Additional lower bands (with 1 or 0 oligosaccharide chains) were observed in the known ALG1-CDG patient (P), who exhibited incomplete glycoforms and was used as the positive control for hypoglycosylation. Serum from the healthy individual (C) was used as the negative control representing the normal TRF glycosylation pattern. ALB and IgG were removed from all samples. (**C**) 2-DE analysis of STD (upper panel) and the son’s serum TRF (lower panel). The numerals indicate the number of terminal sialic acid (Sia) residues on TRF, with each protein spot corresponding to a distinct sialylation level. (**D**) Comparison of TRF isoforms with Sia < 3 and Sia ≥ 3 between the STD and the son’s serum. The gray intensity of protein spots on gel (Figure 2C) was calculated by ImageJ (Version 1.54f, Wayne Rasband, National Institutes of Health, Bethesda, MD, USA). The proportion of Sia < 3 was 6.2% and Sia ≥ 3 was 93.8% in the STD, the proportion of Sia < 3 increased to 14% in the son’s serum TRF.

The siblings had compound heterozygous *ALG1* variants (Figure 1C); namely, c.1129 A>C (maternal) which leads to M377L protein mutant, and the splicing variant c.1263+3 A>T (paternal) whose effects were unknown. ALG1-CDG leads to a specific defect in the assembly of LLO, which is subsequently transferred to the nascent polypeptides in the ER. Consequently, several glycoproteins may be hypoglycosylated, among which serum TRF (Figure 3A) is a model protein [19,29,30,31]. To determine the functional consequences of the *ALG1* variants, sera of the two patients and their parents were tested for potential glycosylation defects (Figure 3A). Western blot analysis revealed that the *N*-glycosylation level of TRF was almost normal in both patients and their parents. Compared with the plasma TRF pattern of the ALG1-CDG positive control, which clearly showed increased hypoglycosylated TRF fractions, the patients’ TRF patterns were similar to that of the healthy control (Figure 3B). In addition, the differences in isoelectric point and molecular weight of different isoforms of TRF were analyzed by IEF (Figure 3C). Intact TRF typically bears four sialic acid residues, while higher branching leads to higher numbers up to six in a small fraction of the protein. Compared with standard human TRF, ALG1-CDG patients exhibited a milder glycosylation phenotype with mono- and disialotransferrin (Figure 3C, lower panel). Calculation of the TRF isoform proportions showed that normal complements (containing 3–6 terminal sialic acid residues) were slightly decreased in the patient (93.8% for STD vs. 86% for the son), while the aberrant complements (containing 0–2 terminal sialic acid residues) were increased (Figure 3D). Therefore, the variants in these patients have a mild effect on Alg1 function and do not significantly affect the *N*-glycosylation pattern.

### 3.4. Plasma TRF Profiling Showed Lower Glycosylation Level and Varied Glycoforms

**Figure 4:**
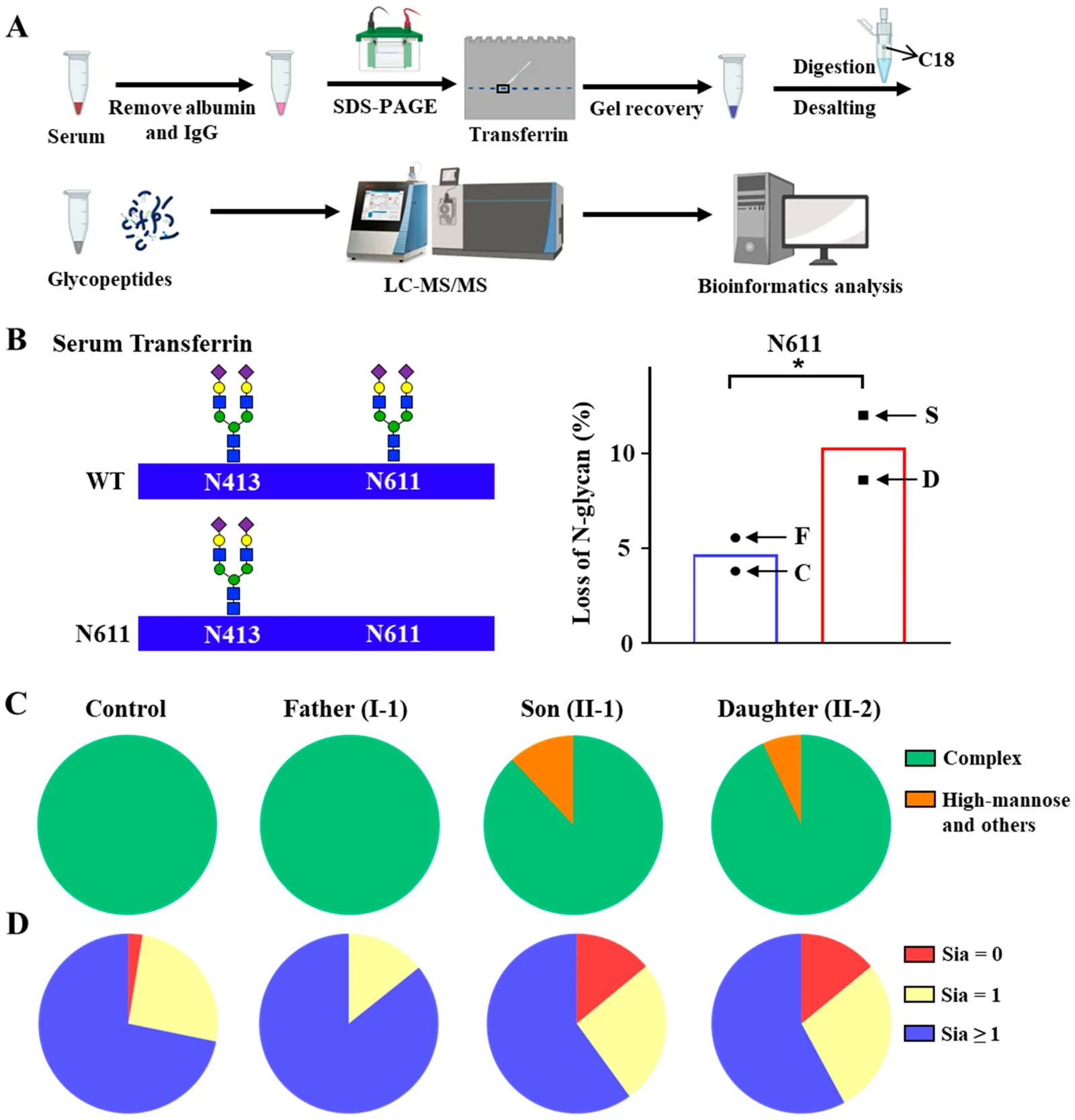
Profiling of the glycopeptides from plasma TRF. (**A**) Flowchart of glycoproteomics experiments. ALB and IgG were removed from all serum samples, which were then separated by 8% SDS-PAGE. After gel excision, the proteins were digested, and the resulting glycopeptides were desalted using a C18 Sep-Pak column. Then, the lyophilized glycopeptides were analyzed using LC-MS/MS. Finally, the acquired MS/MS spectra were profiled using pGlyco3 and MSFragger. (**B**) Analysis of *N*-glycan site occupancy of serum TRF from the healthy control, Father (I-1), Son (II-1) and Daughter (II-2). The healthy control sample was used as the negative control for glycoproteomic comparison, and glycopeptides were identified using MSFragger. *, *p* < 0.05. (**C**) Distribution of different *N*-glycan types on serum TRF in the Control, Father (I-1), Son (II-1) and Daughter (II-2) groups. Glycopeptide data were analyzed by pGlyco3. (**D**) Terminal sialic acid distributions on serum TRF in the Control, Father (I-1), Son (II-1) and Daughter (II-2) groups. The asialo TRF level was higher in the Son (II-1) and Daughter (II-2) than in the Father (I-1) and Control. Glycopeptide data were analyzed by pGlyco3. The search criteria were a precursor tolerance of 10 ppm and a fragment tolerance of 20 ppm. The false discovery rate (FDR) for glycopeptides was 0.01.

TRF has two *N*-glycosylation sites, N413 and N611 [32]. After trypsin digestion, TRF glycopeptides were efficiently enriched and subjected to comprehensive analysis to determine site-specific glycosylation (Figure 4A). Because ALB and IgG are highly abundant proteins in serum, they were removed to reduce their interference with MS identification [15,33]. Representative mass spectra of abnormal glycopeptides identified from the daughter’s serum have been listed in Supplementary Figure S1. Typically, on the glycopeptide IMNGEADAMSLDGGFVYIAGKCGLVPVLAENYN(413)K, there were hypomannosylated (Supplementary Figure S1A) and hybrid-type (Supplementary Figure S1B) *N*-glycans; whereas the glycopeptide QQQHLFGSN(611)VTDCSGNFCLFR carried high-mannose-type *N*-glycans (Supplementary Figure S1C), and non-glycosylated peptides (Supplementary Figure S1D) were also detected. To further address the functional influence of Alg1 variants, glycan occupancy at the glycosylation sites in TRF was analyzed. For the control and patient samples (Figure 4B), glycan occupancy at N611 was slightly but significantly decreased. For the healthy control and father, 3.8% and 5.6% of N611 glycans were lost, respectively, whereas 12% and 8.6% of glycans were lost in the son and daughter, respectively (Figure 4B).

Because TRF mainly bears complex-type sialylated *N*-glycans [30], the glycoforms were subsequently assessed (Figure 4C). Sera from the healthy control and father contained only complex-type *N*-glycans, whereas high-mannose-type and other (e.g., hybrid-type) glycoforms were detected in the serum TRF glycan pools of the son (12%) and daughter (7%). CDG may cause a loss in sialic acid residues from *N*-glycans on TRF [29,30,31,32]. TRF from the healthy control predominantly contained normally assembled *N*-glycans (more than 2 sialic acid residues) and small amounts of intermediate products (28% for the healthy control and 14% for the father) bearing mono- and asialo glycoforms. In contrast, analysis of the profiles of both patients revealed that the level of the normally sialylated glycoforms decreased, accompanied by an accumulation of mono- and asialo glycoforms. Specifically, the abundance of asialylated *N*-glycans increased to 14% in serum TRF from both patients (Figure 4D). These results suggested that the *ALG1* variants in the patients could affect glycoforms involved in *N*-glycan biosynthesis.

### 3.5. ScAlg1 M366L Had Decreased Enzyme Activity

**Figure 5:**
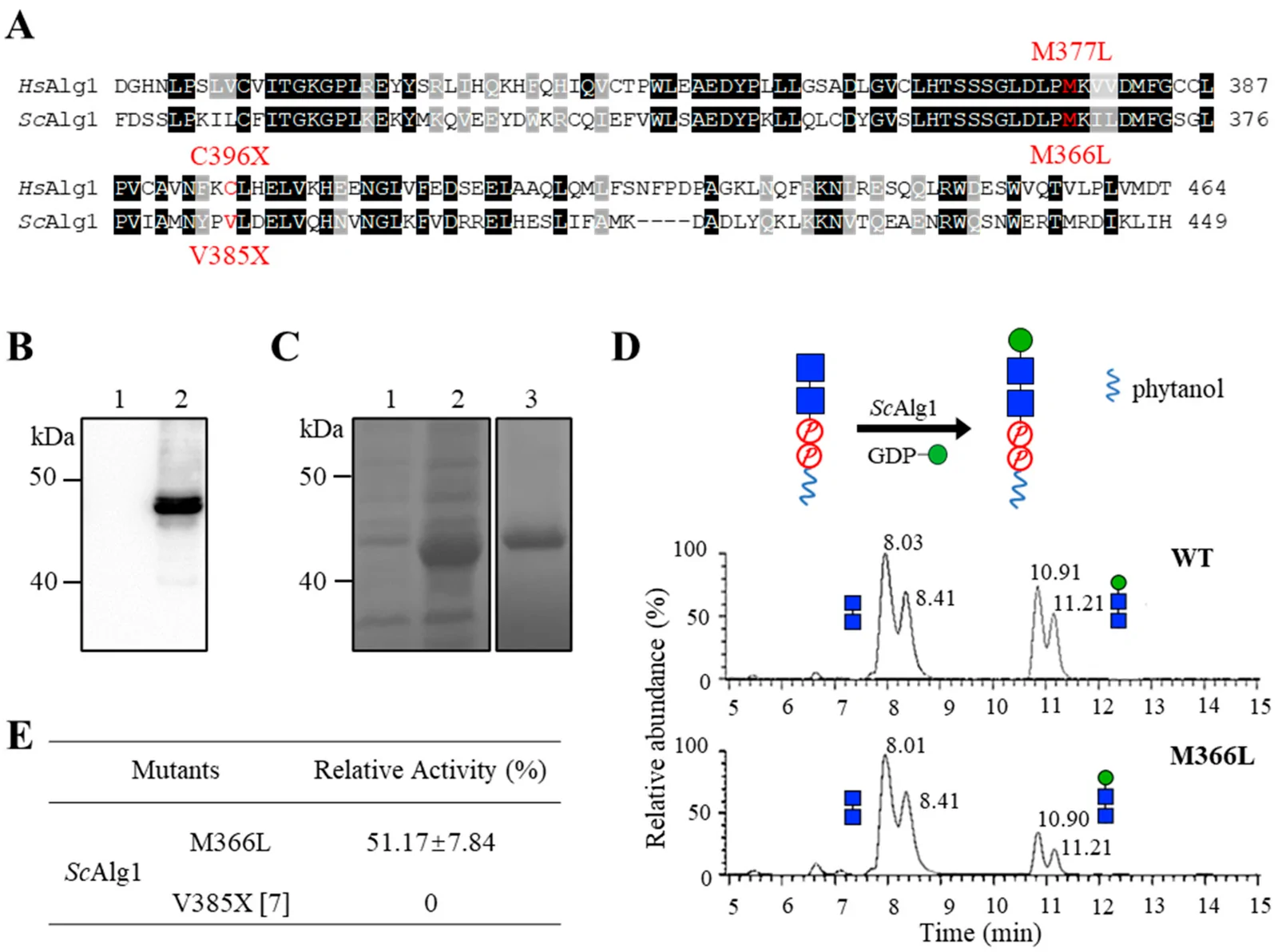
Expression and activity assay of the *Sc*Alg1 M366L mutant. (**A**) Alignment of *Homo sapiens* (*Hs*) and *S. cerevisiae* (*Sc*) Alg1. The conserved amino acids are shaded. Mutations from the patients are indicated by arrows, with human mutations shown above and the corresponding yeast mutants shown below the protein sequence. (**B**) *E. coli* lysates were analyzed by SDS-PAGE and immunoblotted with an anti-His antibody: uninduced *E. coli* (Lane 1) and *E. coli* grown in TB and induced with IPTG (Lane 2). (**C**) SDS-PAGE of purified M366L mutant: uninduced *E. coli* lysate (Lane 1); *E. coli* lysate induced with IPTG (Lane 2); purified *Sc*Alg1 M366L mutant (Lane 3). (**D**) GlcNAc2-PP-Phy was used as the substrate for the reactions catalyzed by purified recombinant *Sc*Alg1 proteins to produce ManGlcNAc2-PP-Phy. The glycans released by acid hydrolysis were subjected to LC-MS analysis for the quantification of the enzymatic activity. UPLC chromatograms of reactions catalyzed by *Sc*Alg1 WT (**upper panel**) and M366L (**bottom panel**) are shown. (**E**) Relative activities of *Sc*Alg1 mutants corresponding to ALG1-CDG mutants. The relative activities of *Sc*Alg1 mutants were calculated relative to wild-type *Sc*Alg1 activity (defined as 100%). The M366L mutant retained 50.88% activity, whereas the C385X mutant showed no detectable activity (0%) as previously reported [7].

The M377L variant corresponded to M366L in *S. cerevisiae* (Figure 5A). To assess the effect of the variant on Alg1 enzyme activity, *Sc*Alg1 M366L, encoding a recombinant His-tagged Alg1 lacking the first 34 amino acids, was expressed in *E. coli*, and the expression level was analyzed by Western blotting using an anti-His antibody. Compared with that in lysates from uninduced control cells, *Sc*Alg1 M366L expression increased markedly after induction with IPTG (Figure 5B). In contrast to the uninduced fraction (Figure 5B, Lane 1), an additional band at 46 kDa was observed after induction, corresponding to the molecular weight of *Sc*Alg1 M366L (Figure 5B, Lane 2). The protein was purified using a HisTrap HP affinity column and subjected to SDS-PAGE (Figure 5C, Lane 3). The yield of purified *Sc*Alg1 M366L was approximately 6 mg per liter of culture.

To assay mannosyltransferase activity, purified proteins were added to a reaction mixture containing GlcNAc2-PP-Phy as the LLO acceptor. Mannosylation of GlcNAc2-PP-Phy was catalyzed by *Sc*Alg1 M366L to produce ManGlcNAc2-PP-Phy (Figure 5D). After acid hydrolysis of the lipid chain, the saccharide moieties were purified and subjected to UPLC‒MS for quantitative analysis. As shown in Figure 5D, two groups of peaks detected by UPLC were assigned to GlcNAc2 and ManGlcNAc2, respectively. The conversion rate was calculated from the peak areas of the substrate (8.03 and 8.41 min) and product (10.91 and 11.21 min). Wild-type *Sc*Alg1 was used as the reference control, and its enzymatic activity was defined as 100%. The relative activity of M366L was calculated based on wild-type activity and determined to be 51.17% (Figure 5E). The results demonstrated that patients’ Alg1 mutant inherited from their mother (*Hs*M377L) may retain only approximately half of the wild-type activity. To further evaluate the potential significance of the newly identified *ALG1* variants, we compared them with previously reported *ALG1* variants regarding residual enzymatic activity and clinical manifestations (Supplementary Table S2).

## 4. Discussion

Although the clinical data of the two patients were reported in 2021, a genetic characteristics analysis has not yet been performed [23]. The maternal variant c.1129 A>C leads to the missense mutant protein M377L, which has not been previously reported [16]. Met 377 is a conserved amino acid in eukaryotes, located between two conserved Asp residues that may affect the mannosyltransferase activity. A variant at the same site, M377V, has been recorded in the ClinVar database (http://www.ncbi.nlm.nih.gov/clinvar) and reported to be pathogenic [16]. The paternal variant c.1263+3 A>T was also novel and was predicted to affect splicing because it is located near exon 11 (Figure 1C). To understand the genotype–phenotype correlation of this intronic variant, the detailed analyses of the biological consequences of splicing variant were performed. The results confirmed that c.1263+3 A>T may provoke skipping of exons 12 and 13, as the transcripts containing exon 1–11 were found in both the father’s and son’s samples. This truncated transcript leads to the C396X protein mutant, which was reported in a previous case of ALG1-CDG and was pathogenic [16]. To evaluate the impact of the c.1263+3 A>T variant on the patient phenotype, the distribution of patient *ALG1* cDNA counts was analyzed (Figure 2A–D). Although not all mature mRNAs inherited from the father were affected by the intronic variant, the C396X variant accounted for up to 73% of the 2612 amplicons, whereas only 16% were normal sequences, which may explain why the patient developed ALG1-CDG at the molecular level. According to ACMG guidelines, Wang et al. classified both c.1129 A>C (PM2+PM3+PM5+PP3+PP4) and c.1263+3 A>T (PM2+PM3+PM5+PP3+PP4) as likely pathogenic [23]. This study further revealed that c.1263+3 A>T may lead to p.C396X, a pathogenic variant; therefore, the ACMG classification should be revised to pathogenic (PVS1+PS3+PM2+PP3).

Serum glycoprotein hypoglycosylation is a common in patients with CDG, and TRF which has two *N*-glycosylation sites, is the most widely used glycoprotein for diagnosis [17,34]. Western blot analysis of TRF was performed using the sera from a healthy control, a parent, two siblings and a patient diagnosed with ALG1-CDG. Surprisingly, unlike most ALG1-CDG patients [9,12,13,16,21,34], the siblings showed an almost normal glycosylation pattern on TRF (Figure 3). In a previous report, three ALG1-CDG patients with M377V and C396X mutants showed similar TRF pattern, in which *N*-glycosylation was mostly normal with slightly hypoglycosylated glycoforms [16]. The clinical and biochemical variability of ALG1-CDG may be associated with the residual activity of different *ALG1* variants. As summarized in Supplementary Table S2, previously reported variants showed diverse clinical outcomes. For example, the p.S258L variant was associated with a severe phenotype and early death [12], whereas the p.M377V variant affecting the same conserved residue as our M377L variant showed relatively preserved glycosylation and residual Alg1 activity [16], although affected individuals still exhibited clinical abnormalities. Consistent with these observations, our M377L variant retained approximately 50% of Alg1 enzymatic activity, which may contribute to the relatively mild glycosylation abnormalities observed in our patients. The molecular basis of this phenomenon is that *ALG1* pathogenic variants in these patients did not completely block *N*-glycan assembly, leaving sufficient enzyme activity to produce nearly normal glycoproteins. Subsequently, TRF *N*-glycans were profiled by LC-MS/MS to further investigate the glycosylation disorder in the siblings (Figure 4). The overall *N*-glycosylation site occupancy was decreased in the TRF of 2 patients, particularly at the N611 site, which lost more than 10% of *N*-glycans (Figure 4B). This may be attributed to the aberrant LLO resulting from the abnormal Alg1, which cannot be transferred by oligosaccharyltransferase to the nascent peptides, thereby leading to underglycosylated TRF secreted outside the cells. Furthermore, the downstream effects of ALG1-CDG in the Golgi were examined. The patients’ TRF exhibited a number of unusual glycoforms, including high-mannose-type and hybrid-type structures, rather than exclusively complex-type *N*-glycans. Subsequently, analysis of the terminal sialic acid distribution of TRF revealed higher levels of asialo- and monosialo *N*-glycans in ALG1-CDG patients than in healthy controls (Figure 4D). Although not critical, the accumulation of asialo- and monosialoglycans indicated *N*-glycan-processing defects in the patients. It is hypothesized that the pathogenic Alg1 mutants in the siblings produced not only nearly normal glycans, but also a small amount of LLO intermediates with incomplete structures. These small intermediates appear to be partially transported into and trimmed in the Golgi, resulting in reduced levels of mature complex-type TRF. These glycoproteomic findings may provide insights into other Alg-related CDG cases.

The pathogenicity of two variants in our study (M377L and C396X) was confirmed by an in vitro mannosyltransferase assay (Figure 5). Natural substrate GlcNAc2-PP-Dol is difficult to synthesize, therefore we established an MS-based in vitro enzymatic assay using phytanyl- instead of dolichol-linked GlcNAc2 (GlcNAc2-PP-Phy) as the acceptor substrate [7]. Because yeast has long served as a canonical eukaryotic model for studying conserved metabolic pathways such as *N*-glycosylation, *S. cerevisiae* Alg1 (*Sc*Alg1) and corresponding counterparts were used to assess the activity of Alg1 mutants from the ALG1-CDG patients. Sequence alignment of Alg1 proteins from *S. cerevisiae* and *H. sapiens* demonstrated that M366L and V385X in yeast correspond to M377L and C396X in humans, respectively (Figure 5A). A homozygous variant (M377V) at this conserved site has been previously reported. In our previous work, *Sc*Alg1 M366V retained only 40% of wild-type activity in the quantitative assay, which was consistent with the mild CDG symptoms of the patient [7]. Here, the recombinant *Sc*Alg1 M366L retained approximately 50% of wild-type activity, indicating partially defective mannosyltransferase activity (Figure 5D). In contrast, the truncated mutant V385X exhibited no transferase activity in our study [7]. For the father, although C396X might be completely inactive, he retained a wild-type *ALG1* allele, resulting in the absence of CDG symptoms. In the siblings, the maternal variant M366L retained approximately half of its activity, whereas only 16% wild-type protein was derived from the paternal allele. Taken together, the patients’ Alg1 activity as calculated from the in vitro assay suggested a 60–70% decrease, which correlates with the clinical severity of their CDG phenotypes [23]. Although the in vitro yeast system provides a robust and evolutionarily conserved platform for evaluating relative mannosyltransferase activity, we acknowledge that extrapolating these specific activities may not fully reflect the absolute activity of human Alg1. Furthermore, other studies have reported patients with nonconserved variants [9,14,16,17], limiting the generalizability of this approach. Accordingly, the estimated activities should be interpreted as comparative rather than absolute indicators of pathogenicity, although they remain consistent with the patients’ clinical presentations. For genetic diseases, it is important to investigate the molecular mechanisms, to understand the genotype–phenotype correlations, and to provide genetic counseling. Our results confirmed the effects of ALG1-CDG variants on enzyme activity, which were related to the patients’ symptoms, suggesting a potential clinical diagnostic approach for ALG1-CDG.

## 5. Conclusions

In summary, this study characterized two novel *ALG1* gene variants identified in two Chinese patients with ALG1-CDG and analyzed their genotypic and phenotypic features. The findings indicate altered glycosylation site occupancy and glycoform profiles in the affected individuals, while functional assays of the missense and splicing variants confirmed their pathogenic effects. Although the conclusions are limited by the small number of patients, they provide valuable insights into the molecular mechanisms underlying ALG1-CDG and enhance our understanding of how mutant Alg1 enzyme activity correlates with disease pathogenicity and clinical severity.

## Data Availability

The datasets used and/or analyzed during the current study are available from the corresponding author upon reasonable request.

## Supplementary Materials

The following supporting information can be downloaded at: https://www.mdpi.com/article/10.3390/biom16091308/s1, Figure S1: Typical MS2 spectra of abnormal glycopeptides identified from the daughter’s serum; Figure S2: Uncropped WB/SDS-PAGE images; Table S1: Primers for PCR amplifications; Table S2: Reported *ALG1* variants and the novel variants identified in this study.

## Abbreviations

The following abbreviations are used in this manuscript:

CDG	Congenital disorders of glycosylation
ALG1-CDG	ALG1-related CDG
SMRT	Single-molecule real-time
GlcNAc2-PP-Dol	Dolichyl-pyrophosphate-GlcNAc2
LLO	Lipid-linked oligosaccharide
RT-PCR	Reverse transcription polymerase chain reaction
CCSs	Circular consensus sequences
SDS-PAGE	Sodium dodecyl sulfate–polyacrylamide gel electrophoresis
ECL	Enhanced chemiluminescence
IEF	Isoelectric focusing
TRF	Transferrin
STD	TRF standard
2-DE	Two-dimensional electrophoresis
IPG	Immobilized pH gradient
CHAPS	3-[(3-Cholamidopropyl) dimethylammonio]-1-propanesulfonate
DTT	Dithiothreitol
BPB	Bromophenol blue
IAM	Iodoacetamide
ACN	Acetonitrile
FA	Formic acid
FDR	False discovery rate
TB	Terrific Broth
GlcNAc2-PP-Phy	Phytanyl-pyrophosphate-GlcNAc2
WT	Wild-type

## References

[1] Francisco R., Brasil S., Poejo J., Jaeken J., Pascoal C., Videira P.A., Ferreira V.D. (2023). Congenital disorders of glycosylation (CDG): State of the art in 2022. Orphanet J. Rare Dis..

[2] Jaeken J., Matthijs G. (2007). Congenital disorders of glycosylation: A rapidly expanding disease family. Annu. Rev. Genom. Hum. Genet..

[3] Ng B.G., Freeze H.H. (2018). Perspectives on Glycosylation and Its Congenital Disorders. Trends Genet..

[4] Wilson M.P., Matthijs G. (2021). The evolving genetic landscape of congenital disorders of glycosylation. Biochim. Biophys. Acta-Gen. Subj..

[5] Ondruskova N., Cechova A., Hansikova H., Honzik T., Jaeken J. (2021). Congenital disorders of glycosylation: Still “hot” in 2020. Biochim. Biophys. Acta-Gen. Subj..

[6] Lehle L., Strahl S., Tanner W. (2006). Protein glycosylation, conserved from yeast to man: A model organism helps elucidate congenital human diseases. Angew. Chem.-Int. Ed..

[7] Li S.T., Wang N., Xu S., Yin J., Nakanishi H., Dean N., Gao X.D. (2017). Quantitative study of yeast Alg1 beta-1, 4 mannosyltransferase activity, a key enzyme involved in protein *N*-glycosylation. Biochim. Biophys. Acta-Gen. Subj..

[8] Wang N., Li S.T., Xiang M.H., Gao X.D. (2022). Alg mannosyltransferases: From functional and structural analyses to the lipid-linked oligosaccharide pathway reconstitution. Biochim. Biophys. Acta-Gen. Subj..

[9] Grubenmann C.E., Frank C.G., Hülsmeier A.J., Schollen E., Matthijs G., Mayatepek E., Berger E.G., Aebi M., Hennet T. (2004). Deficiency of the first mannosylation step in the *N*-glycosylation pathway causes congenital disorder of glycosylation type Ik. Hum. Mol. Genet..

[10] Rohlfing A.K., Rust S., Reunert J., Tirre M., Du Chesne I., Wemhoff S., Meinhardt F., Hartmann H., Das A.M., Marquardt T. (2014). ALG1-CDG: A new case with early fatal outcome. Gene.

[11] Haeuptle M.A., Hennet T. (2009). Congenital Disorders of Glycosylation: An Update on Defects Affecting the Biosynthesis of Dolichol-Linked Oligosaccharides. Hum. Mutat..

[12] Kranz C., Denecke J., Lehle L., Sohlbach K., Jeske S., Meinhardt F., Rossi R., Gudowius S., Marquardt T. (2004). Congenital disorder of glycosylation type Ik (CDG-Ik): A defect of mannosyltransferase I. Am. J. Hum. Genet..

[13] Schwarz M., Thiel C., Lübbehusen J., Dorland B., de Koning T., von Figura K., Lehle L., Körner C. (2004). Deficiency of GDP-Man:GlcNAc_2_-PP-dolichol mannosyltransferase causes congenital disorder of glycosylation type Ik. Am. J. Hum. Genet..

[14] Xue Y., Zhao Y.R., Wu B., Shu J.B., Yan D.D., Li D., Yu X.L., Cai C.Q. (2023). A novel variant in *ALG1* gene associated with congenital disorder of glycosylation: A case report and short literature review. Mol. Genet. Genom. Med..

[15] Zhang W.Y., James P.M., Ng B.G., Li X.L., Xia B.Y., Rong J., Asif G., Raymond K., Jones M.A., Hegde M. (2016). A Novel *N*-Tetrasaccharide in Patients with Congenital Disorders of Glycosylation, Including Asparagine-Linked Glycosylation Protein 1, Phosphomannomutase 2, and Mannose Phosphate lsomerase Deficiencies. Clin. Chem..

[16] Öncül Ü., Kose E., Eminoğlu F.T. (2022). ALG1-CDG: A Patient with a Mild Phenotype and Literature Review. Mol. Syndromol..

[17] Ng B.G., Shiryaev S.A., Rymen D., Eklund E.A., Raymond K., Kircher M., Abdenur J.E., Alehan F., Midro A.T., Bamshad M.J. (2016). ALG1-CDG: Clinical and Molecular Characterization of 39 Unreported Patients. Hum. Mutat..

[18] Barba C., Darra F., Cusmai R., Procopio E., Vici C.D., Keldermans L., Vuillaumier-Barrot S., Lefeber D.J., Guerrini R., Cdg G. (2016). Congenital disorders of glycosylation presenting as epileptic encephalopathy with migrating partial seizures in infancy. Dev. Med. Child Neurol..

[19] Medrano C., Vega A., Navarrete R., Ecay M.J., Calvo R., Pascual S.I., Ruiz-Pons M., Toledo L., García-Jiménez I., Arroyo I. (2019). Clinical and molecular diagnosis of non-phosphomannomutase 2 *N*-linked congenital disorders of glycosylation in Spain. Clin. Genet..

[20] Köse M., Köse E., Kagnici M., Tekin H.G., Özen B., Özdemir T.R., Kirbiyik Ö., Onay H., Yilmaz Ü., Ünalp A. (2020). Congenital Disorder of Glycosylation: Clinical and Molecular Characteristics of 9 Patients from Turkey. Izmir Dr. Behcet Uz Cocuk Hastan. Derg..

[21] González-Domínguez C.A., Fiesco-Roa M.O., Gómez-Carmona S., Kleinert-Altamirano A.P.I., He M., Daniel E.J.P., Raymond K.M., Abreu-González M., Manrique-Hernández S., González-Jaimes A. (2021). ALG1-CDG Caused by Non-functional Alternative Splicing Involving a Novel Pathogenic Complex Allele. Front. Genet..

[22] Bosnyak I., Sadek M., Ranatunga W., Kozicz T., Morava E. (2024). Normal transferrin glycosylation does not rule out severe ALG1 deficiency. J. Inherit. Metab. Dis. Rep..

[23] Wang Q.H., Zou L.P., Wang Y.Y., Shen Y.W., Shi X.Y. (2021). *ALG1* gene-related congenital disorders of glycosylation with infantile spasm: Report of 2 cases and literature review. J. Clin. Pediatr..

[24] Eid J., Fehr A., Gray J., Luong K., Lyle J., Otto G., Peluso P., Rank D., Baybayan P., Bettman B. (2009). Real-time DNA sequencing from single polymerase molecules. Science.

[25] Wen P.P., Chen J.R., Zuo C.Y., Gao X.D., Fujita M., Yang G.L. (2022). Proteome and Glycoproteome Analyses Reveal the Protein *N*-Linked Glycosylation Specificity of STT3A and STT3B. Cells.

[26] Chen W.Y., Wang R., Li D., Zuo C.Y., Wen P.P., Liu H.L., Chen Y.Q., Fujita M., Wu Z.M., Yang G.L. (2020). Comprehensive Analysis of the Glycome and Glycoproteome of Bovine Milk-Derived Exosomes. J. Agric. Food Chem..

[27] Polasky D.A., Yu F., Teo G.C., Nesvizhskii A.I. (2020). Fast and comprehensive *N*- and *O*-glycoproteomics analysis with MSFragger-Glyco. Nat. Methods.

[28] Zeng W.F., Cao W.Q., Liu M.Q., He S.M., Yang P.Y. (2021). Precise, fast and comprehensive analysis of intact glycopeptides and modified glycans with pGlyco3. Nat. Methods.

[29] Adamowicz M., Ploski R., Rokicki D., Morava E., Gizewska M., Mierzewska H., Pollak A., Lefeber D.J., Wevers R.A., Pronicka E. (2007). Transferrin hypoglycosylation in hereditary fructose intolerance: Using the clues and avoiding the pitfalls. J. Inherit. Metab. Dis..

[30] Bengtson P., Ng B.G., Jaeken J., Matthijs G., Freeze H.H., Eklund E.A. (2016). Serum transferrin carrying the xeno-tetrasaccharide NeuAc-Gal-GlcNAc2 is a biomarker of ALG1-CDG. J. Inherit. Metab. Dis..

[31] Park J.H., Weissensteiner M., Wagner O., Wada Y., Rust S., Reunert J., Marquardt T. (2016). Congenital nephrotic syndrome with dysmorphic features and death in early infancy: Answers. Pediatr. Nephrol..

[32] Bruneel A., Cholet S., Tran N.T., Mai T.D., Fenaille F. (2020). CDG biochemical screening: Where do we stand?. Biochim. Biophys. Acta-Gen. Subj..

[33] Abu Bakar N., Ashikov A., Brum J.M., Smeets R., Kersten M., Huijben K., Keng W.T., Speck-Martins C.E., Carvalho D.R., Rizzo I. (2022). Synergistic use of glycomics and single-molecule molecular inversion probes for identification of congenital disorders of glycosylation type-1. J. Inherit. Metab. Dis..

[34] Bogdanska A., Lipinski P., Szymanska-Rozek P., Jezela-Stanek A., Rokicki D., Socha P., Tylki-Szymanska A. (2021). Clinical, biochemical and molecular phenotype of congenital disorders of glycosylation: Long-term follow-up. Orphanet J. Rare Dis..

