# Supplementary material for: Glycoproteomics and Functional Characterization of Novel Variants in Siblings with ALG1-CDG
Source: Biomolecules. 2026 Sep 9;16(9):1308. doi: 10.3390/biom16091308 (PMC13604089; doi:10.3390/biom16091308)
Supplement: Supplementary file 1 [file biomolecules-16-01308-s001.zip › biomolecules-4454149-supplementary.pdf]

Supporting Information

Glycoproteomics and Functional Characterization of Novel Variants in Siblings with ALG1-CDG

Dong-Zhi Wei[1,2†], Hui Wang[1,3†], Sen-Lin Peng[1,2], Yu Huang[4], Ganglong Yang[1,2], Weijie Dong[3], Xiao-Dong Gao[1]* and Ning Wang[1,2]*

[1]State Key Laboratory of Biopharmaceutical Preparation and Delivery, Institute of Process Engineering, Chinese Academy of Sciences, Beijing 100190, China

[2]Key Laboratory of Carbohydrate Chemistry and Biotechnology, Ministry of Education, School of Biotechnology, Jiangnan University, Wuxi 214122, China

[3]College of Basic Medical Sciences, Dalian Medical University, Dalian 116044, Liaoning, China

[4]Department of Medical Genetics, School of Basic Medical Sciences, Peking University, Beijing, China

[†] These authors contributed equally to this work.

* Correspondence authors: wangning@ipe.ac.cn (N. Wang)

1. Supplementary Figures

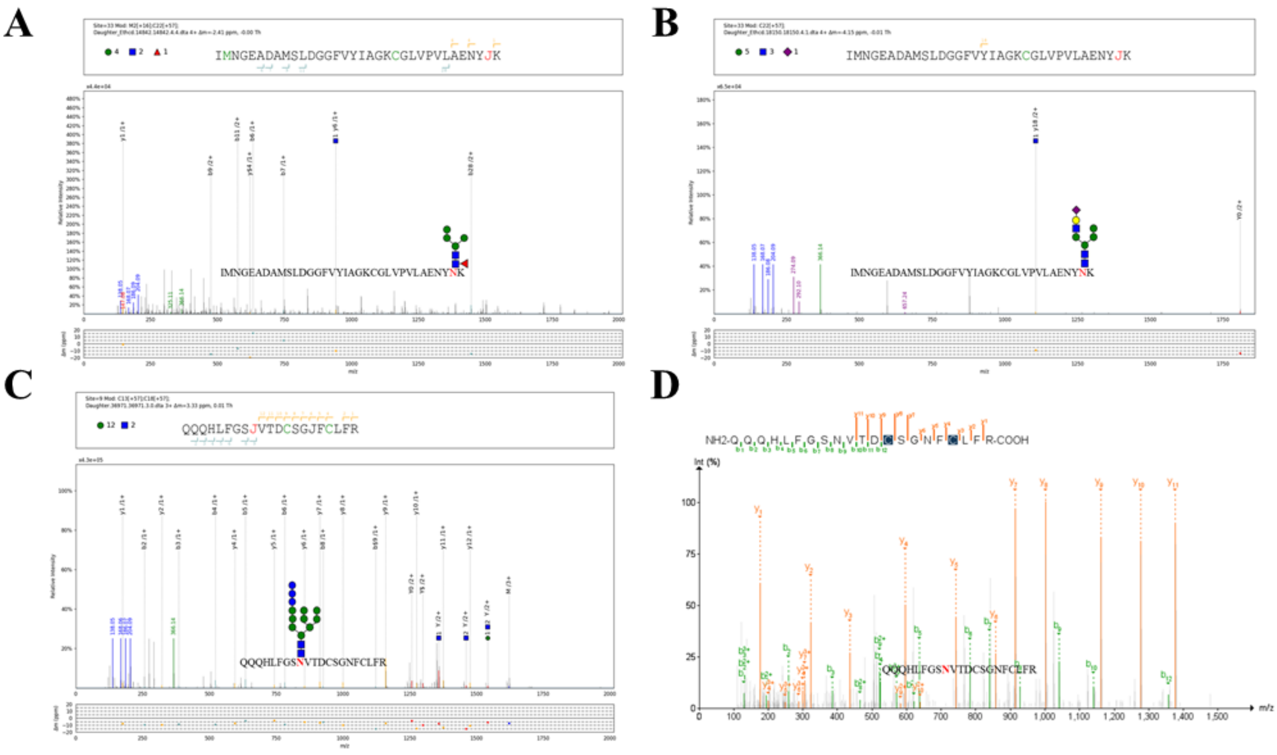

Figure S1 Typical MS2 spectra of abnormal glycopeptides identified from the daughter's serum. Glycopeptides IMNGEADAMSLDGGFVYIAGKCGLVPVLAENYN(413)K-(Hex$_5$HexNAc$_3$Neu5Ac$_1$) (A), IMNGEADAMSLDGGFVYIAGKCGLVPVLAENYN(413)K-(Hex$_4$HexNAc$_2$Fuc$_1$) (B) and QQQHLFGSN(611)VTDCSGNFCLFR-(Hex$_{12}$HexNAc$_2$) (C) were identified by pGlyco3. The peptide QQQHLFGSN(611)VTDCSGNFCLFR lacking glycosylation modification (D) was identified by MSFragger.

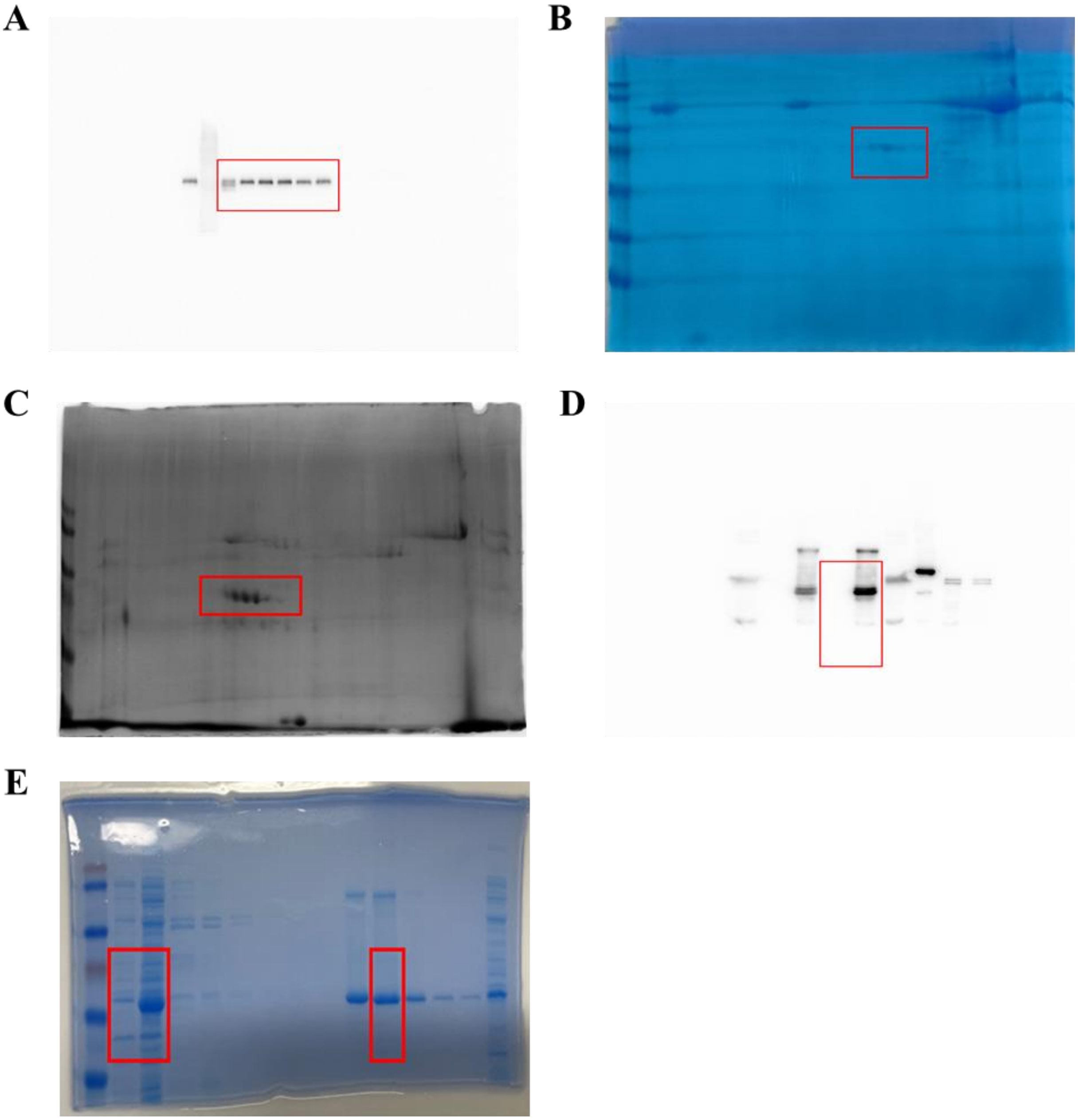

Figure S2 Uncropped WB/SDS-PAGE images. (A) Uncropped WB image of Figure 3B. (B) Uncropped SDS-PAGE image of Figure 3C upper panel. (C) Uncropped SDS-PAGE image of Figure 3C lower panel. (D) Uncropped WB image of Figure 5B. (E) Uncropped SDS-PAGE image of Figure 5C.

2. Supplementary Tables

Table S1 Primers for PCR amplifications

Primer	5' 3' Sequence	PCR product size (bp)
M13F	CGCCAGGGTTTTCCCAGTCACGAC	1550/1262
M13R	AGCGGATAACAATTTCACACAGGA	
P1F	GAATTCAGATTGTGGGGTTGACAGAA	1226/1150
P1R	TTTGCCCTGAGACTCCAAGAAGAA	
SMF	TGGACCTGCCCCTGAAGGTGGTGGAC	6541
SMR	GTCCACCACCTTCAGGGGCAGGTCCA	

Table S2 Reported ALG1 variants and the novel variants identified in this study

	P1	P2	P3	P4	P5
ALG1 mutation	c.773C>T<br>p.S258L	c.1129A>G<br>p.M377V	c.1263G>A; c.1312C>T<br>p.C396X; p.R438W	c.1187+3A>G; c.544G>A<br>/; p.E182K	c.1129 A>C; c.1263+3 A>T<br>p.M377L; p.C396X
Variant type	Missense	Missense	Nonsense; Missense	Splice-site; Missense	Nonsense; Missense
Functional effect /Residual activity	Reduced ALG1 activity; Impaired yeast complementation; Associated with defective glycosylation	Reduced ALG1 function; Predicted damaging variant	Reduced ALG1 function; Predicted loss-of-function	Reduced ALG1 activity; Downstream glycoprotein effects; Glycomic changes	Reduced ALG1 activity; Glycomic changes
Clinical phenotypes	Neonatal seizures; Muscular hypotonia; Cerebral atrophy; Nephrotic syndrome; Progressive neurological impairment	Feeding difficulties; Central hypotonia; Psychomotor retardation; Dysmorphic feature; Normal MRI	Feeding difficulties; Central hypotonia; Multifocal epilepsy; Abnormal MRI; progressive cerebellar hypoplasia; Dysmorphic feature	Left sensorineural hearing loss; Epileptic spasms; Optic nerve atrophy; Hypoplastic brainstem; Cerebellar vermis hypoplasia; Abnormal MRI; Muscle hypotonia	Clinical features in this study
Age at death	11 weeks	Survival	4 years 9 months	Survival	Survival
Reference	[12]	[16]	[16]	[22]	This study
